# Early mortality factors in immune checkpoint inhibitor monotherapy for advanced or metastatic non-small cell lung cancer

**DOI:** 10.1007/s00432-022-04215-7

**Published:** 2022-07-24

**Authors:** Eiji Takeuchi, Kensuke Kondo, Yoshio Okano, Michihiro Kunishige, Yoshihiro Kondo, Naoki Kadota, Hisanori Machida, Nobuo Hatakeyama, Keishi Naruse, Hirokazu Ogino, Hiroshi Nokihara, Tsutomu Shinohara, Yasuhiko Nishioka

**Affiliations:** 1 Department of Clinical Investigation, National Hospital Organization Kochi Hospital, 1-2-25 Asakuranishimachi, Kochi, Kochi 780-8507 Japan; 2 Department of Respiratory Medicine and Rheumatology, Graduate School of Biomedical Sciences, Tokushima University, 3-18-15 Kuramoto-cho, Tokushima, 770-8503 Japan; 3 Department of Respiratory Medicine, National Hospital Organization Kochi Hospital, 1-2-25 Asakuranishimachi, , Kochi, Kochi 780-8507 Japan; 4 Department of Pathology, National Hospital Organization Kochi Hospital, 1-2-25 Asakuranishimachi, Kochi, Kochi 780-8507 Japan; 5 Department of Community Medicine for Respirology, Graduate School of Biomedical Sciences, Tokushima University, 3-18-15 Kuramoto-cho, Tokushima, 770-8503 Japan

**Keywords:** Early death, Early mortality factors, Immune checkpoint inhibitors, Lung cancer, Monotherapy

## Abstract

**Purpose:**

Immune checkpoint inhibitors (ICI) are a promising treatment, but may cause hyperprogressive disease and early death. The present study investigated early mortality factors in ICI monotherapy for lung cancer.

**Patients and methods:**

We retrospectively reviewed all patients diagnosed with advanced or metastatic non-small cell lung cancer (NSCLC) and treated with ICI monotherapy (nivolumab, pembrolizumab, and atezolizumab) between March 2016 and August 2021 at National Hospital Organization Kochi Hospital and Tokushima University. Early death was defined as patients who died within 60 days of ICI treatment.

**Results:**

A total of 166 patients were included. The majority of patients (87%) had an Eastern cooperative oncology group (ECOG) Performance status (PS) of 0/1. There were 21 early deaths. Significant differences were observed in ECOG PS, the histological type, liver metastasis, tumor size, the white blood cell count, neutrophils (%), lymphocytes (%), the neutrophil-to-lymphocyte ratio in serum (sNLR), C-reactive protein (CRP), and albumin between the groups with or without early death. Univariate logistic regression analyses identified ECOG PS score ≥ 2, liver metastasis, tumor size ≥ 5 cm, neutrophils ≥ 69%, lymphocytes < 22%, sNLR ≥ 4, CRP ≥ 1 mg/dl, and albumin < 3.58 g/dl as significant risk factors for early death. A multivariate logistic regression analysis revealed that liver metastasis (Odds ratio [OR], 10.3; *p* = 0.008), ECOG PS score ≥ 2 (OR, 8.0; *p* = 0.007), and a smoking history (OR, 0.1; *p* = 0.03) were significant risk factors for early death.

**Conclusion:**

Liver metastases, ECOG PS score ≥ 2, and a non-smoking history are early mortality factors in ICI monotherapy for advanced or metastatic NSCLC.

## Introduction

Immune checkpoint inhibitors (ICI) have caused a paradigm shift in the treatment of various cancer types. ICI monotherapy has been used as a first-line treatment for advanced or metastatic untreated non-small cell lung cancer (NSCLC) (Reck et al. 2016; Mok et al. 2019). Among patients with advanced, previously treated NSCLC, overall survival (OS) was shown to be significantly better with ICI monotherapy than with chemotherapy (Borghaei et al. 2015; Brahmer et al. 2015; Herbst et al. 2016; Rittmeyer et al. 2017). On the other hand, ICI have been implicated in the development of hyperprogressive disease (HPD) and early death (Champiat et al. 2017; Abbar et al. 2021; Ferrara et al. 2018). Although the risk factors for and mechanisms underlying HPD have been examined (Lo Russo et al. 2019; Kim et al. 2020, 2021; Castello et al. 2020; Passaro et al. 2021; Chen et al. 2021; Ku et al. 2021; Kamada et al. 2019), current knowledge is still insufficient. The selection of suitable patients for ICI monotherapy is essential. OS is the most robust indicator of cancer treatment outcomes. On the other hand, few studies have examined early mortality factors in ICI monotherapy for advanced or metastatic lung cancer in real-world settings. To the best of our knowledge, only one study investigated early death in real-world settings (Inoue et al. 2018). Therefore, the present study attempted to identify early mortality factors in ICI monotherapy for advanced or metastatic NSCLC.

## Materials and methods

### Patients

We retrospectively reviewed all patients diagnosed with advanced or metastatic NSCLC and treated with ICI monotherapy (nivolumab, pembrolizumab, and atezolizumab) between March 2016 and August 2021 at National Hospital Organization Kochi Hospital and Tokushima University. Early death was defined as patients who died within 60 days of ICI treatment.

### Data collection

We collected data on age, sex, smoking history, the Eastern cooperative oncology group performance scale (ECOG PS), white blood cell count, neutrophil count, lymphocyte count, eosinophil count, C-reactive protein (CRP), albumin, histological type, the genotypes of mutations, type of ICI, line of ICI, date of ICI initiation, number of ICI administered, and the status of death. We also examined data obtained on the primary lesion size (maximum diameter measured on chest computed tomography), the number of metastatic sites (count of involved solid organs, not all sites), the status of specific metastasis (non-regional lymph nodes, contralateral lung, pleura, brain, liver, kidney, adrenal gland, and bone), and stage (according to the eighth edition of the tumor-node-metastasis [TNM] staging system). A computed tomography scan was performed for a radiological evaluation before ICI therapy.

### Statistical analysis

Categorical and continuous variables were summarized using descriptive statistics. The independent samples *t* test was used to test for differences between continuous variables. Pearson’s chi-squared test and Fisher’s exact test were employed to analyze relationships between categorical variables. Univariate and multivariate logistic regression analyses were performed to identify predictive factors associated with early death with ICI. The neutrophil-to-lymphocyte ratio in serum (sNLR) was calculated as the ratio of the neutrophil count to the lymphocyte count. The cut-off value for sNLR was set at 4 based on previous studies (Kim et al. 2019b; Sacdalan et al. 2018). We conducted all statistical analyses using SPSS statistics version 27.0 (IBM, Armonk, USA). *P* values are presented without adjustments for multiple comparisons in an exploratory manner.

## Results

### Patient characteristics

A total of 166 advanced or metastatic NSCLC patients treated with ICI monotherapy were included in the present study. Twenty-one out of 166 patients (13%) died within 60 days. Twenty patients (95%) died early due to the rapid progression of lung cancer. One patient (5%) died of immune-related adverse events, including interstitial lung disease. No patient died from exacerbation of complications or other diseases. The clinical characteristics of enrolled patients are summarized in Table 1. Among 166 patients, mean age at ICI therapy was 69 years, 128 (77%) were male, and 130 (78%) were ex- or current smokers. Most patients (87%) had ECOG PS of 0–1. Twenty-one patients (13%) had postoperative recurrence, 31 (17%) had stage III, and 114 (69%) had stage IV. Ninety-eight patients (59%) exhibited adenocarcinoma histology, while 45 (27%) showed squamous cell carcinoma histology. The expression of PD-L1 was higher than 1% in 80% (132/166) of patients, but was absent in 19% (31/166). Six patients (4%) were harboring epidermal growth factor receptor (EGFR) mutations, two (1%) had the anaplastic lymphoma kinase (ALK) rearrangement, and one each (1%) harbored rearranged during transfection (RET) and ROS proto-oncogene 1 (ROS1) fusion. Forty-five patients (27%) received ICI as the first-line therapy. Sixteen patients (10%) had liver metastasis.

**Table 1:** Characteristics of the study population

		Total	≤ 60 days	> 60 days	*p*
		*n* = 166	*n* = 21	*n* = 145	
Age, y	Mean age, (SD)	69 (9)	68 (10)	69 (9)	0.9*
Sex, *n* (%)	Male	128(77)	14 (67)	114 (79)	0.2***
	Female	38 (23)	7 (33)	31 (21)	
Smoking history, *n* (%)	Yes	130 (78)	13 (61)	117 (81)	0.1***
	No	30 (18)	6 (29)	24 (17)	
	Missing	6 (4)	2 (10)	4(3)	
ECOG PS, *n* (%)	0–1	145 (87)	10 (48)	135 (93)	0.001***
	2–4	21 (13)	11 (52)	10 (7)	
Stage, *n* (%)	Recurrence	21 (13)	2 (10)	19 (13)	0.4***
	III	31 (17)	2 (10)	29 (20)	
	IV	114 (69)	17 (81)	97 (67)	
Histological type, *n* (%)	Adeno	98 (59)	16 (76)	82 (57)	0.047***
	Squamous	45 (27)	1 (5)	44 (30)	
	Others	23 (14)	4 (19)	19 (13)	
PD-L1, *n* (%)	< 1%	31 (19)	5 (24)	26 (18)	0.7***
	≥ 1%	132 (80)	16 (76)	116 (80)	
	Missing	3 (2)	0 (0)	3 (3)	
Driver mutation, *n* (%)	None	151 (91)	19 (90)	132 (91)	0.6***
	EGFR	6 (4)	2 (10)	4 (3)	
	ALK	2 (1)	0(0)	2(1)	
	RET	1 (1)	0 (0)	1 (1)	
	ROS1	1(1)	0 (0)	1 (1)	
	Missing	5 (3)	0 (0)	5 (3)	
Treatment line, *n* (%)	1	45 (27)	4 (19)	41 (28)	0.4**
	> 1	121 (73)	17 (81)	104 (72)	
ICI drug, *n* (%)	Pembrolizumab	130 (78)	17 (81)	113 (78)	0.9***
	Nivolumab	35 (21)	4 (19)	31 (21)	
	Atezolizumab	1 (1)	0 (0)	1 (1)	
Liver metastasis, *n* (%)	No	150 (90)	14 (76)	136 (96)	< 0.001***
	Yes	16 (10)	7 (24)	9 (4)	
Tumor size, mm	Mean, (SD)	43.7 (24.1)	56.3 (25.2)	41.8 (23.5)	0.01*
White blood count, /μL	Mean, (SD)	7229 (3404)	9905 (4657)	6842 (3011)	0.01*
Neutrophils, %	Mean, (SD)	67 (10)	72 (8)	66 (10)	0.004*
Lymphocytes, %	Mean, (SD)	22 (9)	15 (5)	23 (9)	< 0.001*
sNLR, ratio	Mean, (SD)	3.9 (2.5)	5.6 (2.5)	3.7 (2.4)	< 0.001*
Eosinophils, %	Mean, (SD)	3 (3)	4 (5)	3 (3)	0.5*
CRP, mg/dL	Mean, (SD)	2.6 (3.4)	4.9 (4.9)	2.3 (3.1)	0.03*
Alb, g/dL	Mean, (SD)	3.5 (0.7)	2.7 (0.7)	3.6 (0.6)	< 0.001*

*ECOG PS* Eastern cooperative oncology group performance status, *PD-L1* programmed death ligand 1, *EGFR* epidermal growth factor receptor, *ALK* anaplastic lymphoma kinase, *RET* rearrangement during transfection, *ROS1* ROS proto-oncogene 1, *ICI* immune checkpoint inhibitor, *sNLR* the neutrophil-to-lymphocyte ratio in serum, *CRP* C-reactive protein, *Alb* albumin

*Independent samples *t* test, **Fisher’s exact test, ***Chi-squared test

Among the 21 patients who died early, mean age was 68 years, 14 (67%) were male, and thirteen (61%) were ex- or current smokers. Ten patients (48%) had an ECOG PS of 0–1. Two patients (10%) had postoperative recurrence, two (10%) had stage III, and 17 (81%) had stage IV. Sixteen patients (76%) showed adenocarcinoma histology, and one (5%) exhibited squamous cell carcinoma histology. The expression of PD-L1 was higher than 1% in 76% (16/21) of patients, but was absent in 24% (5/21). Two patients (10%) harbored EGFR mutations. Four patients (19%) received ICI as the first-line therapy, and seven (24%) had liver metastasis.

Among the 145 patients without early death, mean age was 69 years, 114 (79%) were male, and 117 (81%) were ex- or current smokers. One hundred and thirty-five patients (93%) had an ECOG PS of 0–1. Nineteen patients (13%) had postoperative recurrence, 29 (20%) had stage III, and 97 (67%) had stage IV. Eighty-two patients (57%) exhibited adenocarcinoma histology, and 44 (30%) showed squamous cell carcinoma histology. The expression of PD-L1 was higher than 1% in 80% of patients (116/145), but was absent in 18% (26/145). Four patients (3%) harbored EGFR mutations, two (1%) had the ALK rearrangement, and one each (1%) harbored RET and ROS1 fusion. Forty-one patients (28%) received ICI as the first-line therapy. Nine (4%) had liver metastasis.

Significant differences were observed in ECOG PS, the histological type, liver metastasis, tumor size, the white blood count, neutrophils (%), lymphocytes (%), sNLR, CRP, and albumin between the groups with or without early death.

### Univariate analysis of factors associated with early death

As shown in Table 2, univariate logistic regression analyses identified ECOG PS score ≥ 2 (Odds ratio [OR], 14.9; 95% CI, 5.1–43.3; *p* < 0.001), liver metastasis (OR, 7.6; 95% CI, 2.4–23.4; *p* < 0.001), tumor size ≥ 5 cm (OR, 2.6; 95% CI, 1.03–6.6; *p* = 0.04), neutrophils ≥ 69% (OR, 3.5; 95% CI, 1.3–9.7; *p* = 0.01), lymphocytes < 22% (OR, 10.1; 95% CI, 2.3–45.3; *p* = 0.002), sNLR ≥ 4 (OR, 4.2; 95% CI, 1.6–11; *p* = 0.004), CRP ≥ 1 mg/dl (OR, 6.6; 95% CI, 1.9–23.4; *p* = 0.003), and Alb < 3.58 g/dl (OR, 3.5; 95% CI, 1.5–7.7; *p* = 0.002) as significant risk factors for early death during ICI monotherapy.

**Table 2:** Univariate analysis of factors associated with early death

Variable	Category	Univariate analysis Odds ratio (95% CI)	*p* value
Smoking history	No	1	0.1
	Yes	0.4 (0.2–1.3)	
ECOG PS	0–1	1	** < 0.001**
	2–4	14.9 (5.1–43.3)	
Histological type	Non-adeno	1	0.1
	Adeno	2.5 (0.9–7.1)	
PD-L1	≥ 1%	1	0.7
	< 1%	1.3 (0.4–3.7)	
Liver metastasis	No	1	** < 0.001**
	Yes	7.6 (2.4–23.4)	
Tumor size	< 5 cm	1	**0.04**
	≥ 5 cm	2.6 (1.03–6.6)	
Neutrophils	< 69%	1	**0.01**
	≥ 69%	3.5 (1.3–9.7)	
Lymphocytes	≥ 22%	1	**0.002**
	< 22%	10.1 (2.3–45.3)	
sNLR	< 4	1	**0.004**
	≥ 4	4.2 (1.6–11)	
Eosinophils	≥ 1.5%	1	0.08
	< 1.5%	2.3 (0.9–5.8)	
CRP	< 1 mg/dl	1	**0.003**
	≥ 1 mg/dl	6.6 (1.9–23.4)	
Alb	≥ 3.58 g/dl	1	**0.002**
	< 3.58 g/dl	3.5 (1.5–7.7)	

*p *values <0.05 are bolded

*ECOG PS* Eastern cooperative oncology group performance status, adeno, adenocarcinoma, *PD-L1* programmed death ligand 1, *sNLR* the neutrophil-to-lymphocyte ratio in serum, *CRP* C-reactive protein, *Alb* albumin

### Multivariate analysis of factors associated with early death

A multivariate logistic regression analysis for the prediction of early death was performed using the following variables: a smoking history, ECOG PS, the histological type, liver metastasis, tumor size, neutrophils, lymphocytes, sNLR, CRP, and albumin (Table 3). A multivariate analysis with a multivariate logistic regression model showed that liver metastasis (OR, 10.3; 95% CI, 1.8–58; *p* = 0.008), ECOG PS score ≥ 2 (OR, 8.0; 95% CI, 1.8–36; *p* = 0.007), and a smoking history (OR, 0.1; 95% CI, 0.01–0.8; *p* = 0.03) were significant risk factors for early death during ICI monotherapy.

**Table 3:** Multivariate analysis of factors associated with early death

Variable	Category	Multivariate analysis Odds ratio (95% CI)	*p* value
Smoking history	No	1	**0.03**
	Yes	0.1(0.01–0.8)	
ECOG PS	0–1	1	**0.007**
	2–4	8.0 (1.8–36)	
Histological type	Non-adeno	1	0.1
	Adeno	3.7 (0.7–18.8)	
Liver metastasis	No	1	**0.008**
	Yes	10.3 (1.8–58)	
Tumor size	< 5 cm	1	0.3
	≥ 5 cm	2.5 (0.5–12.5)	
Neutrophils	< 69%	1	0.7
	≥ 69%	0.7 (0.1–4.5)	
Lymphocytes	≥ 22%	1	0.3
	< 22%	3.5 (0.3–40)	
sNLR	< 4	1	0.8
	≥ 4	1.1 (0.2–6.8)	
CRP	< 1 mg/dl	1	0.2
	≥ 1 mg/dl	5.0 (0.5–48.6)	
Alb	≥ 3.58 g/dl	1	0.1
	< 3.58 g/dl	2.9 (0.7–11.4)	

*p *values <0.05 are bolded

*ECOG PS* Eastern cooperative oncology group performance status, adeno, adenocarcinoma, *sNLR* the neutrophil-to-lymphocyte ratio in serum, *CRP* C-reactive protein, *Alb* albumin

### The number of early mortality factors and percentage of early deaths

**Fig. 1:**
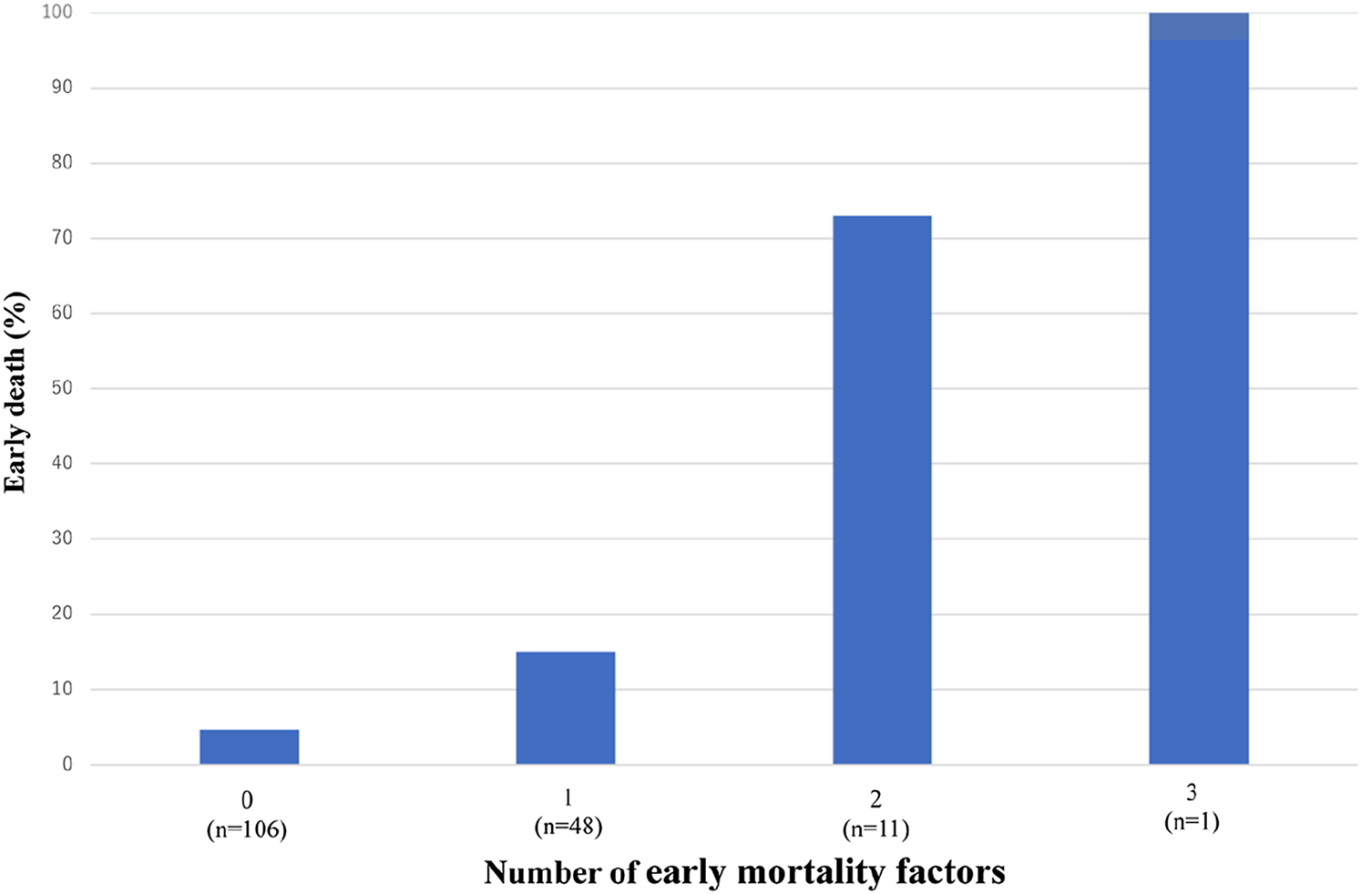
The number of early mortality factors and percentage of early deaths

Liver metastases, ECOG PS score ≥ 2, and a non-smoking history were early mortality factors in ICI monotherapy against advanced or metastatic NSCLC. The number of early mortality factors and percentage of early deaths are shown in Fig. 1. One patient had all three early mortality factors, and the early mortality rate was 100%. Eight out of 11 patients (73%) with two early mortality factors and 7 out of 48 patients (15%) with one early mortality factor died within 60 days. Furthermore, 5 out of 106 patients (4.7%) with no early mortality factors died early.

## Discussion

The present results identified liver metastases, ECOG PS score ≥ 2, and a non-smoking history as early mortality factors in ICI monotherapy for advanced or metastatic NSCLC. Few studies have examined early mortality factors in ICI monotherapy for advanced or metastatic lung cancer in real-world settings. To the best of our knowledge, only one study investigated early mortality in real-world settings (Inoue et al. 2018).

ICI has caused a paradigm shift in the treatment of various cancer types, but have also been implicated in the development of HPD and early patient death (Champiat et al. 2017; Abbar et al. 2021; Ferrara et al. 2018). Although the risk factors for and mechanisms underlying HPD have been examined (Lo Russo et al. 2019; Kim et al. 2020, 2021; Castello et al. 2020; Passaro et al. 2021; Chen et al. 2021; Ku et al. 2021; Kamada et al. 2019), current knowledge is still inadequate. Although PD-L1 and the tumor mutation burden (TMB) have been used as predictive biomarkers for ICI therapy, there are currently no established predictive biomarkers (Brody et al. 2017; Zou et al. 2021). The selection of suitable patients for ICI monotherapy is essential in real-world settings, and OS is the most robust indicator of cancer treatment outcomes. Therefore, we examined early mortality factors in ICI monotherapy for advanced or metastatic NSCLC in real-world settings.

ECOG PS ≥ 2, a pretreatment CRP-to-albumin ratio > 0.3, and poor responses to previous treatment have been associated with early death in ICI monotherapy (Inoue et al. 2018). In the present study, a multivariate analysis with a multivariate logistic regression model identified liver metastasis (OR, 10.3; 95% CI, 1.8–58; *p* = 0.008), ECOG PS score ≥ 2 (OR, 8.0; 95% CI, 1.8–36; *p* = 0.007), and a smoking history (OR, 0.1; 95% CI, 0.01–0.8; *p* = 0.03) as risk factors for early death in ICI monotherapy. ECOG PS ≥ 2 is frequently identified as an early mortality factor.

In a system review, liver metastasis, previous metastatic sites > 2, a Royal Marsden Hospital score ≥ 2, higher ECOG PS, and a lactate dehydrogenase (LDH) level greater than the normal upper limit were associated with the development of HPD (Chen et al. 2020). In another system review, liver metastasis, LDH, more than two metastatic sites, and a Royal Marsden Hospital score of 2 or higher correlated with HPD (Kim et al. 2019a). A previous study also implicated hepatic metastases, more than two metastatic sites, ECOG PS ≥ 2, and an LDH level higher than the normal upper limit in the development of HPD (Chen et al. 2021). Furthermore, liver metastasis was the only variable associated with HPD in a multivariate analysis (Abbar et al. 2021). The presence of tumors within the liver was previously shown to significantly reduce systemic tumor-specific immunity, and immunosuppression was dependent on regulatory T cells (Tregs) (Lee et al. 2020). The number of circulating Tregs was significantly higher than the baseline in patients with HPD (Kang et al. 2022). The balanced expression of PD-1 between CD8 + T cells and Tregs is essential in programmed cell death 1 (PD-1) inhibitory immunotherapy (Aksoylar and Boussiotis 2020; Kumagai et al. 2020). In a mouse model of liver metastasis due to colorectal cancer, Treg numbers were elevated, and the hepatocyte growth factor/hepatocyte growth factor receptor signaling pathway was up-regulated (Huang et al. 2020). Lactic acid has been shown to promote the expression of PD-1 in Tregs in highly glycolytic tumors, such as liver metastases (Kumagai et al. 2022).

In the present study, a non-smoking history was a significant early mortality factor. OR was 0.1 (*P* = 0.03) when six patients with a missing smoking history were assumed to have a smoking history and 0.2 (*P* = 0.03) when they were hypothesized to have a non-smoking history, indicating that non-smoking history is a risk factor regardless of the presence or absence of missing data. The reason for this result is not apparent. In adenocarcinoma, a non-smoking status was predictive of a low TMB (Sharpnack et al. 2020). The probability of EGFR mutations and ALK rearrangements was previously shown to be significantly higher in patients with a non-smoking history than in those with a smoking history (Chapman et al. 2016). Furthermore, TMB was lower in EGFR-mutant lung cancer than in EGFR wild-type lung cancer (Offin et al. 2019). EGFR mutations and ALK rearrangements are generally associated with low response rates to ICI therapy in NSCLC (Gainor et al. 2016; Lee et al. 2017). Therefore, ICI may be less effective in patients with a non-smoking history. However, in the present study, only 2 out of 21 patients who died early had EGFR mutations and no ALK rearrangement. No significant differences were observed in genetic abnormalities between the groups with and without early death (Table 1). Specific molecular alterations (MDM2/MDM4, KRAS, and serine/threonine kinase 11) have been associated with HPD (Kato et al. 2017; Kim et al. 2019b). Therefore, these genetic abnormalities in patients with a non-smoking history may be related; however, only a few have been examined to date.

The limitation of the present study is that it was a two-center retrospective analysis conducted with heterogeneous data from patient cohorts. Therefore, the results obtained are speculative and not definitive. Furthermore, since the number of cases was small, OR was significant, but CI was also large; therefore, reliability was not high. The frequency of early death within 60 days of ICI monotherapy for advanced or metastatic NSCLC was not high. Therefore, multicenter studies are needed. Although we need to consider these limitations when interpreting the present results, this study is of value because early mortality factors in ICI monotherapy against advanced or metastatic NSCLC were identified in real-world settings. Simple clinical parameters may easily predict early mortality with single-agent ICI against advanced or metastatic NSCLC and are clinically useful. Early mortality rates were 73 and 100% in patients with 2 and 3 early mortality factors, respectively. ICI-based combination therapy may be a better treatment option for patients with multiple mortality factors than ICI monotherapy for advanced or metastatic NSCLC.

## Conclusion

Liver metastases, ECOG PS score ≥ 2, and a non-smoking history were identified as early mortality factors in ICI monotherapy against advanced or metastatic NSCLC.

## Data Availability

None.

## Abbreviations

ALK: Anaplastic lymphoma kinase
CI: Confidence interval
CRP: C-reactive protein
ECOG: Eastern Cooperative oncology group
EGFR: Epidermal growth factor receptor
HPD: Hyperprogressive disease
ICI: Immune checkpoint inhibitors
LDH: Lactate dehydrogenase
NSCLC: Non-small cell lung cancer
PD-1: Programmed cell death 1
PD-L1: Programmed death ligand 1
OR: Odds ratio
OS: Overall survival
PS: Performance status
RET: Rearranged during transfection
ROS1: ROS proto-oncogene 1
Tregs: Regulatory T cells
sNLR: Neutrophil-to-lymphocyte ratio in serum
TMB: Tumor mutation burden
